# COVID-19 pandemic and STEMI: pathway activation and outcomes from the pan-London heart attack group

**DOI:** 10.1136/openhrt-2020-001432

**Published:** 2020-10-26

**Authors:** Callum D Little, Tushar Kotecha, Luciano Candilio, Richard J Jabbour, George B Collins, Asrar Ahmed, Michelle Connolly, Ritesh Kanyal, Ozan M Demir, Lucy O Lawson, Brian Wang, Sam Firoozi, James C Spratt, Divaka Perera, Philip MacCarthy, Miles Dalby, Ajay Jain, Simon J Wilson, Iqbal Malik, Roby Rakhit

**Affiliations:** 1 Department of Cardiology, Royal Free London NHS Foundation Trust, London, United Kingdom; 2 Institute of Cardiovascular Science, University College London, London, United Kingdom; 3 Department of Cardiology, Imperial College Healthcare NHS Trust, London, United Kingdom; 4 Department of Cardiology, Barts Health NHS Trust, London, United Kingdom; 5 Department of Cardiology, Royal Brompton & Harefield NHS Foundation Trust, London, United Kingdom; 6 Department of Cardiology, St George’s University Hospitals NHS Foundation Trust, London, United Kingdom; 7 Department of Cardiology, King’s College Hospital NHS Foundation Trust, London, United Kingdom; 8 Department of Cardiology, Guy’s and St Thomas’ NHS Foundation Trust, London, United Kingdom

**Keywords:** percutaneous coronary intervention, acute coronary syndrome, chest pain, myocardial infarction

## Abstract

**Objectives:**

To understand the impact of COVID-19 on delivery and outcomes of primary percutaneous coronary intervention (PPCI). Furthermore, to compare clinical presentation and outcomes of patients with ST-segment elevation myocardial infarction (STEMI) with active COVID-19 against those without COVID-19.

**Methods:**

We systematically analysed 348 STEMI cases presenting to the PPCI programme in London during the peak of the pandemic (1 March to 30 April 2020) and compared with 440 cases from the same period in 2019. Outcomes of interest included ambulance response times, timeliness of revascularisation, angiographic and procedural characteristics, and in-hospital clinical outcomes

**Results:**

There was a 21% reduction in STEMI admissions and longer ambulance response times (87 (62–118) min in 2020 vs 75 (57–95) min in 2019, p<0.001), but that this was not associated with a delays in achieving revascularisation once in hospital (48 (34–65) min in 2020 vs 48 (35–70) min in 2019, p=0.35) or increased mortality (10.9% (38) in 2020 vs 8.6% (38) in 2019, p=0.28). 46 patients with active COVID-19 were more thrombotic and more likely to have intensive care unit admissions (32.6% (15) vs 9.3% (28), OR 5.74 (95%CI 2.24 to 9.89), p<0.001). They also had increased length of stay (4 (3–9) days vs 3 (2–4) days, p<0.001) and a higher mortality (21.7% (10) vs 9.3% (28), OR 2.72 (95% CI 1.25 to 5.82), p=0.012) compared with patients having PPCI without COVID-19.

**Conclusion:**

These findings suggest that PPCI pathways can be maintained during unprecedented healthcare emergencies but confirms the high mortality of STEMI in the context of concomitant COVID-19 infection characterised by a heightened state of thrombogenicity.

**Key questions**

**What is already known about this subject?**

- The rates of ST-segment elevation myocardial infarction (STEMI) admissions worldwide have decreased during the COVID-19 pandemic.
- Single-centre data suggest disruption to emergency services may result in delays to the primary percutaneous coronary intervention (PPCI) pathway including time from pain onset to first medical contact and in-hospital delivery of revascularisation.
- The systemic inflammatory response, induced by COVID-19, appears to disrupt antithrombotic mechanisms contributing to a higher incidence of thrombotic complications. The implications for acute coronary syndromes remain unclear.

**What does this study add?**

- Consistent with international findings there has been a 21% decrease in STEMI cases in London during the COVID-19 pandemic compared with the same period in 2019.
- Modifications to the existing in-hospital PPCI pathways, such as routine use of personal protective equipment and redeployment of staff to other clinical areas, do not result in delayed time taken to achieve coronary revascularisation (door to balloon time) or worse clinical outcomes
- Ambulance response times have increased during the pandemic period, likely due to the high volume of COVID-19-illness related calls.
- Patient with COVID-19 and STEMI have a significantly higher coronary thrombus burden compared with COVID-19 negative patients.

**Key questions**

**How might this impact on clinical practice?**

- A targeted antithrombotic approach may be of benefit in these patients but further prospective studies are required.
- Systemic thrombolysis has been suggested as an alternative strategy to managing patients with STEMI during the pandemic; however, our data suggest that even at the peak of COVID-19 related admissions to hospital, it has been possible to maintain effective PPCI services.

## Introduction

The current COVID-19 global pandemic has required urgent restructuring of established clinical pathways in order to manage the surge of patients presenting with SARS-CoV-2. As of 17 July 2020, there have been more than 13 million confirmed cases of COVID-19 worldwide and 584 940 deaths.^1^ In the UK, daily admissions to hospital with COVID-19 peaked at 3260 on 1 April 2020.^2^

Additionally, COVID-19 poses a risk for healthcare workers, necessitating routine use of personal protective equipment (PPE) and modifications to existing medical services and pathways.^3^ In particular, managing patients with suspected or proven COVID-19 represents a significant risk to the delivery of a primary percutaneous coronary intervention (PPCI) pathway where time-dependent revascularisation is key to successful outcomes.^4^ It has been suggested that the current pandemic may result in delays to the pathway including time from pain onset to first medical contact and in-hospital delivery of revascularisation.^3^

Moreover, COVID-19 may have a significant impact on presentation, angiographic findings^5 6^ and clinical outcomes of patients presenting with ST-segment elevation myocardial infarction (STEMI) through the PPCI pathway.^7^ The systemic inflammatory response, induced by COVID-19, appears to disrupt antithrombotic mechanisms.^8^ Furthermore, cellular viral inclusions and the resultant inflammation may produce endothelial injury,^9^ thrombotic microangiopathy^10^ and microvascular dysfunction further potentiating a procoagulopathic state and contributing to the higher incidence of thrombotic complications identified in these patients.^11^

## Aims

To evaluate the effect of the COVID-19 pandemic on an established ambulance-triggered PPCI programme involving seven high-volume heart attack centres in London, UK. Data and outcomes from 2020 were compared with the same time period from 2019. Furthermore, we sought to compare clinical presentation and outcomes of patients with STEMI with active COVID-19 against those of patients with STEMI without COVID-19.

## Methods

### Study design and patient population

The PPCI programme in London is the largest urban network of seven heart attack centres in the UK using a single ambulance triggered service and providing 24/7 treatment for STEMI to a population of 9 million. We conducted a retrospective observational analysis of consecutive PPCI pathway activations to all seven heart attack centres in London, UK. The study period was 1 March to 30 April 2020, corresponding with the peak of daily reported COVID-19 cases in the UK. A control period of 1 March to 30 April 2019 was used for comparison. Of patients presenting via the PPCI pathway during the study period, we included those with (1) an ECG consistent with STEMI^4^; and (2) a culprit infarct-related lesion on coronary angiography requiring intervention. Patients who did not present via the PPCI pathway, such as those self-presenting to hospital or those developing STEMI as an inpatient were not included. Patients who underwent coronary angiography revealing unobstructed coronary vessels and/or those who were given an alternative diagnosis were excluded. Data were collected from the local British Cardiac Intervention Society (BCIS) databases. We conducted two distinct analyses, first comparing data of the 2020 study period with a 2019 control group, and second within the 2020 cohort comparing patients with confirmed COVID-19 to non-COVID-19 patients.

### BCIS-National Institute for Cardiovascular Outcomes Research Database

The BCIS-National Institute for Cardiovascular Outcomes Research Database collects data from all hospitals performing PCI in UK.^12^ Data are collected prospectively at each hospital, electronically encrypted and transferred online to a central database. Patients’ survival data are obtained by linkage of patients’ National Health Service numbers to the Office of National Statistics.

### COVID-19 status

COVID-19 positive status was defined as either (1) the presence of a positive oro/nasopharyngeal throat swab for SARS-CoV-2 by reverse-transcriptase PCR; or (2) a clinical diagnosis based on a combination of typical symptoms, radiographic appearances^13^ and characteristic blood test parameters, as per the European Centre for Disease Prevention and Control criteria.^14^ Those who did not meet these criteria were deemed COVID-19 negative.

### PPCI pathway and procedural characteristics

PPCI pathway timings and procedural characteristics were recorded in all procedural PPCI reports, as part of the UK National Cardiac Audit Programme.^15^ We included four time points: (1) symptom onset (pain time); (2) first call to emergency services for medical assistance (call time); (3) arrival at PPCI centre (door time); (4) first coronary intervention restoring perfusion to infarct-related artery (IRA, balloon time). Total ischaemic time was defined as the period from symptoms onset to balloon time.

Procedural characteristics of interest included: vessel(s) attempted; total number of vessels attempted (n); total number of lesions attempted (n); total number of stents inserted (n); total length of stent used (mm); widest diameter balloon used (mm); use of glycoprotein IIb/IIIa inhibitor; use of aspiration thrombectomy and ‘thrombolysis in myocardial infarction’ (TIMI) flow grade^16^ in the IRA at the end of the case.

Presentations following out-of-hospital cardiac arrest or with cardiogenic shock were identified. Cardiogenic shock was defined in the BCIS registry as persistent hypotension with clinical evidence of hypoperfusion (cool, clammy, oliguric, altered mental status) with dependence on inotropes or mechanical left ventricular support to correct this situation.^17^

### Clinical characteristics and outcomes

All-cause mortality during STEMI-related hospitalisation, admission to an intensive care unit (ICU) and total length of inpatient stay (days) were determined from electronic patient records and discharge summaries. In addition, baseline demographic characteristics and admission blood tests including high-sensitivity troponin-T, creatinine, ferritin, haemoglobin, C reactive protein (CRP) and lymphocyte count were also retrieved.

### Patient/public involvement statement

There was no involvement of patients and the public in this study

### Statistical analysis

Data analysis was performed using GraphPad Prism V.7.00 (GraphPad Software, La Jolla, California, USA). Continuous data were presented as a mean±SD or median (IQR) and compared using Student’s t test (parametric) or Mann-Whitney U (non-parametric). Normality was assessed using the Kolmogorv-Smirnov test. Categorical data were presented as numbers with percentages and compared using Pearson’s χ^2^ test and OR (95% CI). A p value of <0.05 was deemed to be of statistical significance.

## Results

A total of 788 patients fulfilled the inclusion criteria (348 during the 2020 study period and 440 patients during the 2019 control period). There was a 21% decrease in STEMI presentations in 2020 (incident rate ratio 0.79). Four patients in the 2020 study period received upfront systemic thrombolysis as the initial reperfusion strategy but still required bailout PPCI. No patient in the 2019 control period received systemic thrombolysis. Within the 2020 group, 46 patients (13.2%) fulfilled the criteria of COVID-19 infection and 302 were COVID-19 negative.

### Effect of the pandemic on clinical pathways: 2020 versus 2019 cohort

**Figure 1:**
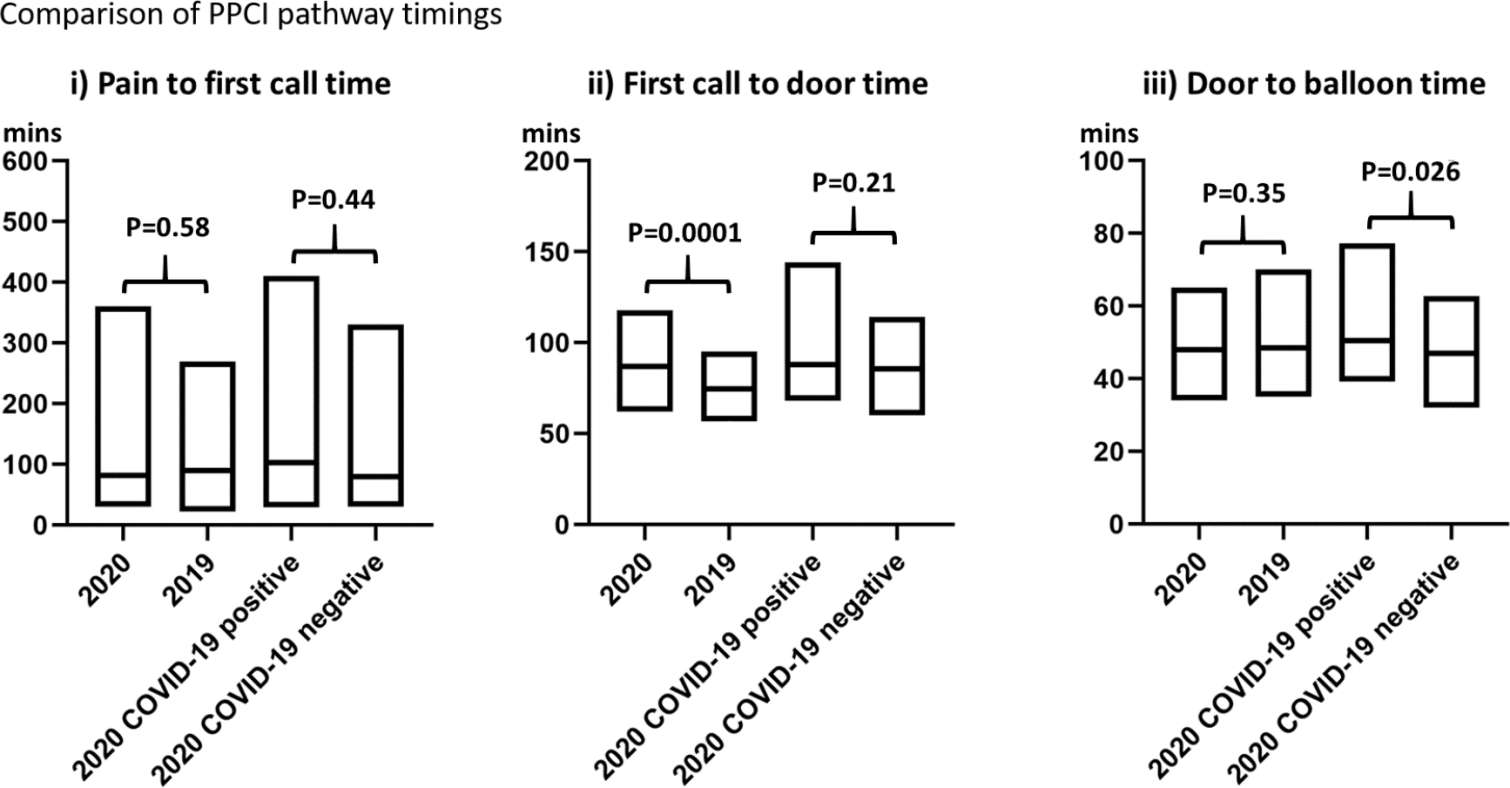
Pathway data are displayed as an IQR (box plot) and median (bold horizontal line within the box plot).

No significant difference was observed between the 2020 and 2019 cohorts with regards to baseline demographics (table 1) and procedural characteristics (table 2). Aspiration thrombectomy and rates of cases completed with TIMI flow less than 3 were similar between both groups (respectively, 19.5% (68) vs 20.9% (92), p=0.64, and 9.5% (33) vs 7.1% (31), p=0.21). There was no significant difference in pain to first call for help (82 (30–360) min in 2020 vs 90 (22–269) min in 2019, p=0.58) or door to balloon (48 (34–65) min in 2020 vs 48 (35–70) min in 2019, p=0.35). First call to door time was significantly longer in the 2020 cohort compared with the 2019 cohort (87 (62–118) min vs 75 (57–95) min, p<0.001; figure 1). There was no significant difference in ICU admission (10.6% (37) in 2020 vs 9.8% (43) in 2019, p=0.69) or in-hospital all-cause mortality (10.9% (38) in 2020 vs 8.6% (38) in 2019, p=0.28; table 2).

**Table 1:** Comparison of ST-segment elevation myocardial infarction (STEMI) admissions: 2020 study cohort versus 2019 control cohort: demographic and primary percutaneous coronary intervention (PPCI) pathway characteristics

	2020 study cohort	2019 control cohort	P value	OR (95% CI)
	(n=348)	(n=440)		
*Baseline demographic characteristics*				
Age (years)	63 (55–71)	63 (55–73)	0.36	
Male sex	278 (80%)	343 (78%)	0.51	
Diabetes	86 (24.7%)	106 (24.1%)	0.87	
Hypertension	178 (51.2%)	217 (49.3%)	0.61	
Hyperlipidaemia	124 (35.6%)	154 (35%)	0.85	
Smoking	145 (41.6%)	245 (55.7%)	<0.001*	
Previous myocardial infarction	43 (12.4%)	60 (13.6%)	0.61	
Stroke	11 (3.2%)	18 (4.1%)	0.53	
Previous PCI	43 (12.4%)	63 (14.3%)	0.42	
Previous CABG	4 (1.2%)	12 (2.7%)	0.12	
Peripheral vascular disease	10 (2.9%)	8 (1.8%)	0.32	
Renal disease	12 (3.5%)	13 (3%)	0.69	
Family history of IHD	41 (11.8%)	65 (14.8%)	0.22	
*PPCI pathway characteristics*				
Pain—First call (min)	82 (30–360)	90 (22–269)	0.58	
First call—Door (min)	87 (62–118)	75 (57–95)	0.001*	
Door—Balloon (min)	48 (34–65)	48 (35–70)	0.35	
Total ischaemic time (min)	282 (173–618)	246 (157–536)	0.049*	
Out of hospital cardiac arrest	10.8% (38)	8.9% (39)	0.36	1.24 (0.78 to 1.98)

Baseline demographic characteristics and PPCI pathway timings of patients with STEMI admitted during the study periods in 2020 versus 2019.

*Denotes statistical significance (p<0.05).

**Table 2:** Comparison of ST-segment elevation myocardial infarction (STEMI) admissions: 2020 study cohort versus 2019 control cohort: procedural characteristics and clinical endpoints

	2020 study cohort	2019 control cohort	P value	OR (95% CI)
	(n=348)	(n=440)		
*Procedural characteristics*				
Lesions treated	1 (1–1)	1 (1–1)	0.15	
Vessels treated	1 (1–1)	1 (1–1)	0.81	
Stents	1 (1–2)	1 (1–2)	0.57	
Total length of stent (mm)	30 (21–38)	28 (20–38)	0.38	
Widest balloon (mm)	3.5 (3–4)	3.5 (3–4)	0.56	
Cardiogenic shock	47 (13.5%)	55 (12.5%)	0.68	1.09 (0.72 to 1.64)
Gp2b3a inhibitor use	142 (41%)	159 (36%)	0.16	1.23 (0.92 to 1.63)
Thrombus aspiration use	68 (19.5%)	92 (20.9%)	0.64	0.91 (0.65 to 1.30)
TIMI flow <3 at end of case	33 (9.5%)	31 (7.1%)	0.21	1.32 (0.82 to 2.34)
*Clinical endpoints*				
ICU admission	37 (10.6%)	43 (9.8%)	0.69	1.10 (0.69 to 1.73)
Length of stay (days)	3 (2–4)	3 (2–5)	<0.001*	
In-hospital mortality	38 (10.9%)	8.6% (38)	0.28	1.30 (0.81 to 2.08)

Procedural characteristics and clinical endpoints of patients with STEMI admitted during the study periods in 2020 versus 2019.

*Denotes statistical significance (p<0.05).

ICU, intensive care unit; TIMI, thrombolysis in myocardial infarction.

### Clinical presentation and outcomes in 2020: COVID-19 positive versus COVID-19 negative

There was no significant difference in baseline characteristics between the COVID-19 positive and COVID-19 negative cohorts (table 3) with the exception of greater hyperlipidaemia in the COVID-19 positive group (52.2% (24) vs 33.1% (100), p=0.012) and more frequent history of previous PCI in the COVID-19 negative group (2.2% (1) vs 13.3% (40), p=0.03). As expected, there was a statistically significant difference with respect to lower lymphocyte count, elevated ferritin and CRP in the COVID-19 positive group. No difference was seen with respect to high sensitivity Troponin T between the two groups (table 2).

**Table 3:** Comparison of COVID-19 positive ST-segment elevation myocardial infarction (STEMI) versus COVID-19 negative STEMI: baseline demographic characteristics, primary percutaneous coronary intervention (PPCI) pathway characteristics and admission blood tests

	COVID-19 positive (n=46)	COVID-19 negative (n=302)	P value	OR (95% CI)
*Baseline demographic characteristics*				
Age (years)	63 (58–67)	63 (55–72)	0.66	
Male sex	37 (80.4%)	241 (79.8%)	0.66	
Diabetes	15 (32.6%)	71 (23.5%)	0.18	
Hypertension	25 (54%)	153 (50.7%)	0.64	
Hyperlipidaemia	24 (52.2%)	100 (33.1%)	0.012*	
Smoking	19 (41.3%)	126 (41.7%)	0.96	
Previous myocardial infarction	5 (10.9%)	38 (12.6%)	0.74	
Stroke	1 (2.2%)	10 (3.3%)	0.68	
Previous PCI	1 (2.2%)	40 (13.3%)	0.03*	
Previous CABG	0 (0%)	4 (1.3%)	0.43	
Peripheral vascular disease	2 (4.4%)	8 (2.7%)	0.52	
Renal disease	1 (2.2%)	10 (3.3%)	0.68	
Family history of IHD	7 (15.2%)	34 (11.3%)	0.44	
*Admission blood tests*				
Haemoglobin (g/L)	137 (116–151)	141 (129–152)	0.15	
Lymphocytes (×10^9^/L)	1.3 (0.88–1.7)	1.6 (1.2–2.2)	0.003*	
Aspartate aminotransferase (IU/L)	66 (33–217)	86 (30–231)	0.74	
Alanine transaminase (IU/L)	46 (32–65)	32 (22–59)	0.087	
Ferritin (µg/L)	427 (213–1529)	176 (93–313)	<0.001*	
C reactive protein (mg/L)	28 (3–122)	5 (3–23)	0.002*	
Creatinine (µmol/L)	83 (68–96)	81 (68–98)	0.66	
High-sensitivity troponin T (ng/L)	749 (174–2617)	419 (83–2260)	0.18	
*PPCI pathway characteristics*				
Pain—First call (min)	103 (30–410)	80 (30–331)	0.44	
First call—Door (min)	88 (68–144)	86 (60–114)	0.21	
Door—Balloon (min)	51 (39–77)	47 (32–63)	0.026*	
Total ischaemic time (min)	360 (223–1418)	257 (172–580)	0.008*	
Out of hospital cardiac arrest	5 (10.9%)	33 (10.9%)	0.99	0.99 (0.40 to 2.69)

Baseline demographic characteristics, admission blood tests, PPCI pathway timings of patients with STEMI and concurrent COVID-19 and those without COVID-19 admitted during the study periods in 2020.

CABG, coronary artery bypass grafting; IHD, ischaemic heart disease.

With regards to pathway timings (figure 1), there were no statistically significant differences in median time from pain to first call (103 (30–410) min vs 80 (30–331) min, p=0.44) or first call to door (88 (68–144) min vs 86 (60–114) min, p=0.21). Door to balloon time was significantly increased for COVID-19 positive patients (51 (39–77) min vs 47 (32–63) min, p=0.026).

Within recorded procedural characteristics (table 4), in the COVID-19 positive cohort the use of aspiration thrombectomy (30.4% (14) vs 17.9% (54), OR 2.01 (95% CI 0.99 to 4.05), p=0.046) and glycoprotein IIb/IIIa inhibitor use (56.5% (26) vs 38.7% (117), OR 2.06 (95% CI 1.12 to 3.87), p=0.022) was significantly higher. COVID-19 positive patients had significantly higher rates of procedures with a final TIMI flow less than 3 (19.6% (9) vs 8% (24), OR 2.82 (95% CI 1.16 to 6.45), p=0.012). The total length of stent used was higher in the COVID-19 positive group (38 mm (24–48) vs 28 mm (20–38), p=0.012). In addition, COVID-19 positive patients with STEMI had higher rates of postprocedural ICU admission (32.6% (15) vs 9.3% (28) OR 5.74 (95% CI 2.24 to 9.89), p<0.001), in-hospital all-cause mortality (21.7% (10) vs 9.3% (28) OR 2.72 (95% CI 1.25 to 5.82), p=0.012) and longer in-hospital length of stay (4 (3–9) days vs 3 (2–4) days, p<0.001).

**Table 4:** Comparison of COVID-19 positive ST-segment elevation myocardial infarction (STEMI) versus COVID-19 negative STEMI: procedural characteristics and clinical endpoints

	COVID-19 positive (n=46)	COVID-19 negative (n=302)	P value	OR (95% CI)
*Procedural characteristics*				
Lesions treated	1 (1–1)	1 (1–1)	0.44	
Vessels treated	1 (1–1)	1 (1–1)	0.53	
Stents	1 (1–2)	1 (1–2)	0.99	
Total length of stent (mm)	38 (24–48)	28 (20–38)	0.012*	
Widest balloon (mm)	3.5 (2.5–3.75)	3.5 (3–4)	0.14	
Cardiogenic shock	6 (13%)	41 (13.6%)	0.92	0.95 (0.40 to 2.30)
Gp2b3a inhibitor use	26 (56.5%)	117 (38.7%)	0.022*	2.06 (1.12 to 3.87)
Thrombus aspiration use	14 (30.4%)	54 (17.9%)	0.046*	2.01 (0.99 to 4.05)
TIMI flow <3 at end of case	9 (19.6%)	24 (8%)	0.012*	2.82 (1.16 to 6.45)
*Clinical endpoints*				
ICU admission	15 (32.6%)	28 (9.3%)	<0.001*	5.74 (2.24 to 9.89)
Length of stay (days)	4 (3–9)	3 (2–4)	<0.001*	
In-hospital mortality	10 (21.7%)	28 (9.3%)	0.012*	2.72 (1.25 to 5.82)

Procedural characteristics and clinical endpoints of patients with STEMI and concurrent COVID-19 and those without COVID-19 admitted during the study periods in 2020.

ICU, intensive care unit; TIMI, thrombolysis in myocardial infarction.

## Discussion

The global COVID-19 pandemic has resulted in significant disruption to healthcare systems. In particular, there is a growing body of evidence worldwide demonstrating a significant decrease of up to 40% in the volume of patients presenting through PPCI services during the pandemic.^5 18–20^ Our data support these findings with heart attack services in London experiencing a 21% decrease in cases compared with the same period in 2019. There could be a number of explanations for this observation. First, patients experiencing chest pain may have been reluctant to seek medical attention either in fear of viral transmission or not wishing to burden the already stretched healthcare system. Second, patients with no cardiac history may have attributed their myocardial infarction symptoms to COVID-19 infection and, as per government advice at the time, did not seek immediate help. Finally, those patients who did seek help may not have been prioritised by the ambulance service who were under immense pressure at the time and a proportion of these patients may have deteriorated and died before receiving medical attention. In addition to the fall in acute presentations, survey data suggest a corresponding increase in the incidence of cardiac related complications associated with untreated STEMI^21^; however, the long-term implications remain unclear.

The key to an effective PPCI service is minimisation of total ischaemic time.^4^ Critical to this pathway is prompt identification of STEMI, rapid transfer to a PPCI centre and immediate revascularisation on arrival. Delays at any stage during this pathway may increase myocardial ischaemia time resulting in reduced myocardial salvage, larger infarct size and subsequent long term morbidity.^22 23^ COVID-19 resulted in multiple potential delays to this pathway. Our analysis comparing the 2020 study population to the 2019 control population in a busy metropolitan city importantly revealed comparable door to balloon times between the two groups, contrary to previous reports^24^ with comparable in-hospital mortality rates. This suggests that modifications to the existing in-hospital PPCI pathways, such as routine use of PPE and redeployment of staff to other clinical areas, neither delayed the time taken to achieve coronary revascularisation (door to balloon time) or resulted in worse outcomes. However, the median ischaemic time was 36 min longer in 2020 than 2019, driven predominantly by an increase in the median time from first call for help to arrival at a PPCI centre. The likely explanation for this is the impact of a high volume of COVID-19-illness related calls overwhelming paramedic emergency services leading to a delayed response time, although additional time for paramedics to don PPE may also have contributed. The increased door to balloon time and subsequent increased ischaemic time for COVID-19 positive patients compared with COVID-19 negative patients in 2020 likely reflects the increased procedural complexity of these cases rather than system-related delays as all centres mandated full PPE for all PPCI cases given the unknown COVID-19 status at time of procedure. While systemic thrombolysis has been suggested as an alternative strategy to managing patients with STEMI during the pandemic,^3^ our data suggest that even at the peak of COVID-19 related admissions to hospital, it was possible to maintain effective PPCI services.

Current literature suggest that patients with COVID-19 presenting with STEMI have higher rates of in-hospital mortality, compared with those without concurrent COVID-19.^25^ Our data support this finding with more than doubling in the rate of in-hospital mortality for these patients. Furthermore, the increased frequency of ICU admission and longer length of stay have significant implications on hospital resources. Importantly, there was no significant difference in the baseline characteristics between COVID-19 positive and COVID-19 negative patients. The cause of this excess mortality is not clear but it is likely that a number of factors may be contributory. First, COVID-19 itself is associated with high in-hospital mortality and the infarct in addition to systemic illness results in poor prognosis particularly in those with significant comorbidity.^5^ Second, delays in presentation result in longer ischaemic which is known to be associated with larger infarct size and therefore these patients are more likely to develop fatal post-MI complications.^26^ Third, patients with COVID-19 appear to be highly prothrombotic compared with COVID-19 negative patients, with studies demonstrating high incidence of pulmonary thromboembolic disease as well as thrombi in other organs such at the kidneys.^8^ High coronary thrombus burden at presentation is independently associated with increased rates of major adverse cardiovascular events and mortality.^27 28^ Furthermore, intraprocedural distal embolisation of thrombus may disrupt microvascular function resulting in angiographic no reflow and increase infarct size.^29^ Thrombus burden determination is highly subjective and as such we employed thrombus aspiration usage and reduced TIMI flow at the end of the procedure as surrogates. Reduced TIMI flow is frequently seen with distal embolisation of thrombotic material.^30^ This study demonstrates an increased use of thrombus aspiration and glycoprotein IIb/IIIa inhibitors within the COVID-19 cohort, with higher rates of reduced TIMI flow, suggesting a higher thrombotic burden when compared with non-COVID-19 patients with STEMI. Glycoprotein IIb/IIIa inhibitors have a class IIa indication for adjunctive therapy with PCI with evidence of high thrombotic burden^4^ and may be of benefit in patients with COVID-19 although with increased risk of bleeding. A targeted antithrombotic approach may be of benefit in these patients to decrease rates of distal embolisation of thrombus and subsequently decrease infarct size in order to improve long-term outcomes but further prospective studies are required.

Choudry *et al*^6^ have recently published a single centre experience demonstrating a signal of increased thrombus burden in COVID-19 patients with STEMI (n=39) with higher use of aspiration thrombectomy and glycoprotein IIb/IIIa inhibitors, as well as higher postintervention TIMI thrombus grade and lower resultant myocardial blush score. Importantly, our multicentre study includes the largest cohort of COVID-19 positive patients with STEMI (n=46) compared with COVID-19 negative patients with similar presentation during the same time period, and in addition to confirming these findings also demonstrates a significant increase in mortality in the COVID-19 positive group and delays in door to balloon time. Our additional control group of patients with STEMI from 2019 provides key insights into the effects of modifications to existing PPCI pathways. Mafham *et al*^20^ have highlighted a substantial reduction in acute coronary syndrome presentations in England during the COVID-19 pandemic period compared with 2019. Our study provides further analysis based on the presence or absence of concurrent COVID-19 infection and validates their findings by providing details of both the heart attack pathways and procedural characteristics.

Limitations of the current study include its retrospective design in the first instance and therefore the potential introduction of information bias. Additionally, we only included patients receiving PCI for STEMI and data on those subjects with STEMI who did not undergo PCI (including those receiving systemic thrombolysis only and those managed medically in the first instance) were not included in our analysis. Moreover, patients who underwent coronary angiography that revealed no culprit lesion were excluded from the analysis. It has been reported that in COVID-19 positive patients presenting with STEMI between 33% and 39% of cases demonstrate no culprit lesion on angiography.^5 25^ This represents a large proportion of admissions through the PPCI pathway and the effects of pathway modifications on this cohort within our study are not assessed. While this is a large study of PPCI services in a metropolitan city, the COVID-19 positive group was relatively small making up less than 5% of the cohort and therefore findings within this group should be interpreted with caution. Moreover, data are limited to in-hospital outcomes. Long-term prospective follow-up will be required to determine the true association between COVID-19 and excess mortality following STEMI. Furthermore, the threshold used by emergency service to initiate the PPCI pathway, during the pandemic, may have been influenced by factors relating to infection control and resource management. This may have contributed to the reduction seen in PPCI activations worldwide in 2020. Finally, the outcome of those patients who did not present to the PPCI service is unknown. They may have significantly worse late outcomes, with heart failure, arrhythmia and death as yet unmeasured in the community.

## Conclusion

The COVID-19 pandemic has resulted in unprecedented strain on hospital services in London and as a consequence of this, a number of modifications to the existing PPCI pathway were required. While these factors resulted in increased ambulance response times, there was no adverse effect on door-to-balloon times or mortality. This suggests that even under the extreme pressures of increased COVID-19 related ambulance call-outs, delayed response times, routine PPE use and increased procedural complexity, well-established clinical pathways can be maintained without compromising patient outcomes. Second, patients with COVID-19 infection presenting with STEMI exhibit higher thrombotic burden and are at significant increased risk of death.
